# Increased In-Hospital Mortality in Immunocompromised Individuals Hospitalized With COVID-19 During the Global Pandemic: A Multinational Cohort Study in the EuCARE Project

**DOI:** 10.1093/infdis/jiag254

**Published:** 2026-05-08

**Authors:** Ashley O Roen, Pontus Hedberg, Joana P Ventura Pereira, Maurizio Zazzi, Dovile Juozapaite, Milosz Parczewski, João Domingos, Francis Drobniewski, Giulia Marchetti, Luca Carioti, Pontus Nauclér, Anders Sönnerborg, Björn-Erik Ole Jensen, Francesca Incardona, Alessandro Cozzi-Lepri

**Affiliations:** Centre for Clinical Research, Epidemiology, Modelling and Evaluation, Institute for Global Health, University College London, London, United Kingdom; Department of Medicine Huddinge, Karolinska Institutet, Stockholm, Sweden; Department of Gastroenterology, Hepatology and Infectious Diseases, Medical Faculty and University Hospital Duesseldorf, Heinrich Heine University, Duesseldorf, Germany; Department of Medical Biotechnologies, University of Siena, Siena, Italy; Vilnius Santaros Klinikos Biobank, Vilnius University Hospital Santaros Klinikos, Vilnius, Lithuania; Department of Infectious, Tropical Diseases and Acquired Immunodeficiency, Pomeranian Medical University, Szczecin, Poland; Microbiology Laboratory, Department of Infectious Diseases, Unidade Local de Saúde de Lisboa Ocidental, Lisboa, Portugal; Department of Infectious Disease, Imperial College London, London, United Kingdom; Clinic of Infectious Diseases and Tropical Medicine, San Paolo Hospital, ASST Santi Paolo e Carlo, Department of Health Sciences, University of Milan, Milan, Italy; Department of Experimental Medicine, University of Rome Tor Vergata, Rome, Italy; Department of Infectious Diseases, Karolinska University Hospital, Stockholm, Sweden; Division of Infectious Diseases, Department of Medicine Solna, Karolinska Institutet, Stockholm, Sweden; Department of Medicine Huddinge, Karolinska Institutet, Stockholm, Sweden; Department of Infectious Diseases, Karolinska University Hospital, Stockholm, Sweden; Department of Gastroenterology, Hepatology and Infectious Diseases, Medical Faculty and University Hospital Duesseldorf, Heinrich Heine University, Duesseldorf, Germany; EuResist Network GEIE, Via Guido Guinizelli, 98/100, 00152 Rome, Italy; InformaPRO s.r.l., Via Guido Guinizelli, 98/100, 00152 Rome, Italy; Centre for Clinical Research, Epidemiology, Modelling and Evaluation, Institute for Global Health, University College London, London, United Kingdom

**Keywords:** immunocompromising conditions, COVID-19, SARS-CoV-2, in-hospital mortality

## Abstract

**Background:**

Although coronavirus disease 2019 (COVID-19) is no longer a public health emergency, it remains the most prevalent circulating infectious-like illness in Europe. Whether immunocompromising conditions (ICCs) still carry increased mortality risk during the Omicron era is unclear.

**Methods:**

We conducted a cohort study across EuCARE sites in 8 countries among adults admitted to hospital with COVID-19 between 2020 and 2023. ICCs and COVID-19 pneumonia at hospitalization were defined using clinical information and ICD-10 codes. Logistic regression and counterfactual mediation analysis was used to compare 28-day in-hospital mortality risk associated with ICCs using COVID-19 pneumonia and vaccination at hospital entry as intermediates. Proportion of the total effect of ICCs mediated and the controlled direct effects (CDEs) were calculated. We also formally tested for interaction between SARS-CoV-2 variants and ICCs for mortality risk.

**Results:**

A total of 42 488 individuals were included, of whom 1675 (3.9%) had an ICC. Fifty-five percent were male, and the median age was 67 (IQR, 52–79) years. Overall, 4344 (10.2%) individuals died in hospital. ICCs were associated with increased mortality (OR, 1.49 [95% CI, 1.25–1.79]), with no evidence for an attenuation during the Omicron phase (*P* value for interaction = .60). Mediation analyses showed that the total effect of ICCs was mediated by vaccination but only weakly by pneumonia. With Omicron, the excess mortality associated with ICC was higher under the scenario that everyone in the cohort was to develop COVID-19 pneumonia (CDE, 1.22 [95% CI, .09–1.65]).

**Conclusions:**

ICC remains a significant risk factor for in-hospital death, even during the Omicron era, particularly if the infection led to the development of pneumonia.

## Teaser

A study of 42 488 hospitalized COVID-19 patients (2020–2023) found that immunocompromising conditions (ICCs) increased 28-day mortality risk (OR, 1.49), with no evidence for attenuation during Omicron. ICCs remained a key death risk, especially with pneumonia.

Although the World Health Organization declared the end of coronavirus disease 2019 (COVID-19) as a public health emergency in May 2023, severe acute respiratory syndrome coronavirus 2 (SARS-CoV-2) continues to pose a serious threat, and immunocompromised individuals remain at high risk for progression to severe disease [1, 2]. Despite the circulation of less virulent variants and widespread immunization, the burden of disease among immunocompromised patients remains disproportionately high, due to underlying immunodeficiency, immunosuppressive therapies, suboptimal vaccine response [3–5], and the relaxation of public health precautions [6].

The heterogeneity of immunocompromising conditions (ICCs) has led to variability in reported COVID-19 severity. Some studies describe increased hospitalization, severe disease following COVID-19, and reduced vaccine efficacy [7–10], whereas others find no excess mortality risk among solid organ transplant (SOT) recipients [11, 12]. Patients with hematological malignancies (HMs) also face elevated mortality rates and impaired vaccine responses, particularly during B-cell–depleting treatments [13, 14]. Three recent studies analyzing the impact of ICCs throughout the pandemic reported increased mortality among all ICCs, even in the Omicron era characterised by lower degree of pathogenicity [15–17]. However, the first of these recent studies did not directly evaluate the role of vaccination and compared the risk of death in people with ICCs with that of a historical cohort of people with pneumonia [15]. The remaining studies did not have data on pneumonia and showed results of the association with ICCs after controlling for COVID-19 vaccination by regression adjustment. We believe, based on pure temporality arguments, that neither COVID-19 vaccination nor pneumonia are confounders but rather intermediates in the association between ICCs and mortality so that controlling for vaccination may be introducing, instead of removing, bias [16, 17].

In this analysis, we investigate the relationship between immunocompromised status and in-hospital mortality among patients admitted with COVID-19 across different waves of the pandemic and test whether this relationship changed over time. We also assess severe disease during hospitalization (ie, the development of COVID-19 pneumonia) and COVID-19 vaccination as potential mediators in the pathway between ICCs and death. Finally, we evaluate the direct effect of ICCs under counterfactual scenarios in which the whole cohort versus no one in the cohort were to experience one of these intermediates, overall, and after stratification for variants.

Under strong assumptions regarding the causal structure of the data, we aim to clarify the mechanisms underlying the high risk of death in this vulnerable group following SARS-CoV-2 infection and to identify opportunities for targeted interventions to reduce mortality in people with immunosuppression.

## METHODS

### Data

Using data from the European cohorts of patients and schools to advance response to epidemics (EuCARE)-HOSPITALIZED study [18] (ClinicalTrials.gov identifier NCT05463380) within the EuCARE project (www.eucareresearch.eu), we conducted a retrospective cohort study among adults hospitalized with COVID-19 across 10 centers in 8 countries: Italy, Portugal, Germany, United Kingdom, Kenya, Sweden, Lithuania, and Poland. Data were uploaded to a centralized database from each site using electronic health records, health registries, and other sources (see the Supplementary Materials for complete details for each cohort).

### Study Population

Adults (≥18 years of age) hospitalized with COVID-19, defined as testing positive for SARS-CoV-2 by polymerase chain reaction or antigen positivity between 14 days prior to or during hospitalization (but not after hospital discharge), were included. Hospitalizations from 1 March 2020 until 31 April 2023 were considered, covering a wide range of SARS-CoV-2 circulating variants from the wild-type strain until Omicron BA.5. Participants with multiple hospital admissions contributed their first admission to this analysis. Only EuCARE centers providing ICC classification were included in the final analysis.

### Definitions

The ICCs considered were SOT, HM, or hematopoietic stem cell transplantation (HSCT), defined according to the definitions described by *International Classification of Diseases, Tenth Revision, Clinical Modification* (ICD-10) codes Z94.0, Z94.1, Z94.2, Z94.3, Z94.4, Z94.81, and C81–C96. We categorized immunocompromised groups as SOT, HM with HSCT, HM without HSCT, and both HM and SOT. Whether participants carried other less severe ICCs was also recorded, but this information was not used to define the exposure of interest. ICCs were assumed to preexist hospital entry and had to be diagnosed within 12 months of SARS-CoV-2 diagnosis, or the patient had to be receiving treatment for these chronic conditions in the 12 months preceding their SARS-CoV-2 hospitalization.

### Pneumonia

COVID-19 pneumonia at or during hospitalization was defined based on the ICD-10 codes J12.8 and J12.9, or a physician defining it as clinical and/or radiological pneumonia, or patients with newly arising need for supplemental oxygen.

### Vaccination Status

Vaccination status prior to hospitalization was categorized as a binary variable if the individual had at least 2 doses of any SARS-CoV-2 vaccine up until 14 days prior to hospital admission, as this has been shown to decrease hospitalization and death [19]. In sensitivity analyses, we used a threshold of at least 3 doses to define protective vaccination; we also performed a sensitivity analysis requiring the last vaccination date to be within 6 months of hospital admission to account for peak vaccine effectiveness [20]

### Study Outcome and Other Variables

The primary outcome was time to all-cause in-hospital mortality within 28 days, defined as the interval from the date of hospital admission to the date of death. Follow-up was censored at hospital discharge or at 28 days, whichever occurred first. A patient could be discharged home, to another hospital, or to a care facility, but discharge reason was not collected in the study. The variants of SARS-CoV-2 that we included in our analysis were wild-type (B1_D614gG), Alpha (B.1.1.7), Delta (B.1.617.2), Omicron BA.1 (B.1.1.529_B), Omicron BA.2, Omicron BA.4, Omicron BA.5, Omicron BQ.1 (BQ.1.1), and Omicron CH.1 (CH.1.1). Participants with other variants were not included due to low numbers in these categories. For a proportion of participants (15.5%), the SARS-CoV-2 variant was classified using sequencing. If sequencing data were not available, the variant was inferred from the 7-day moving average national distribution of variants retrieved from the Global Initiative on Sharing All Influenza Data (GISAID), as we have done previously [2].

**Figure 1.**
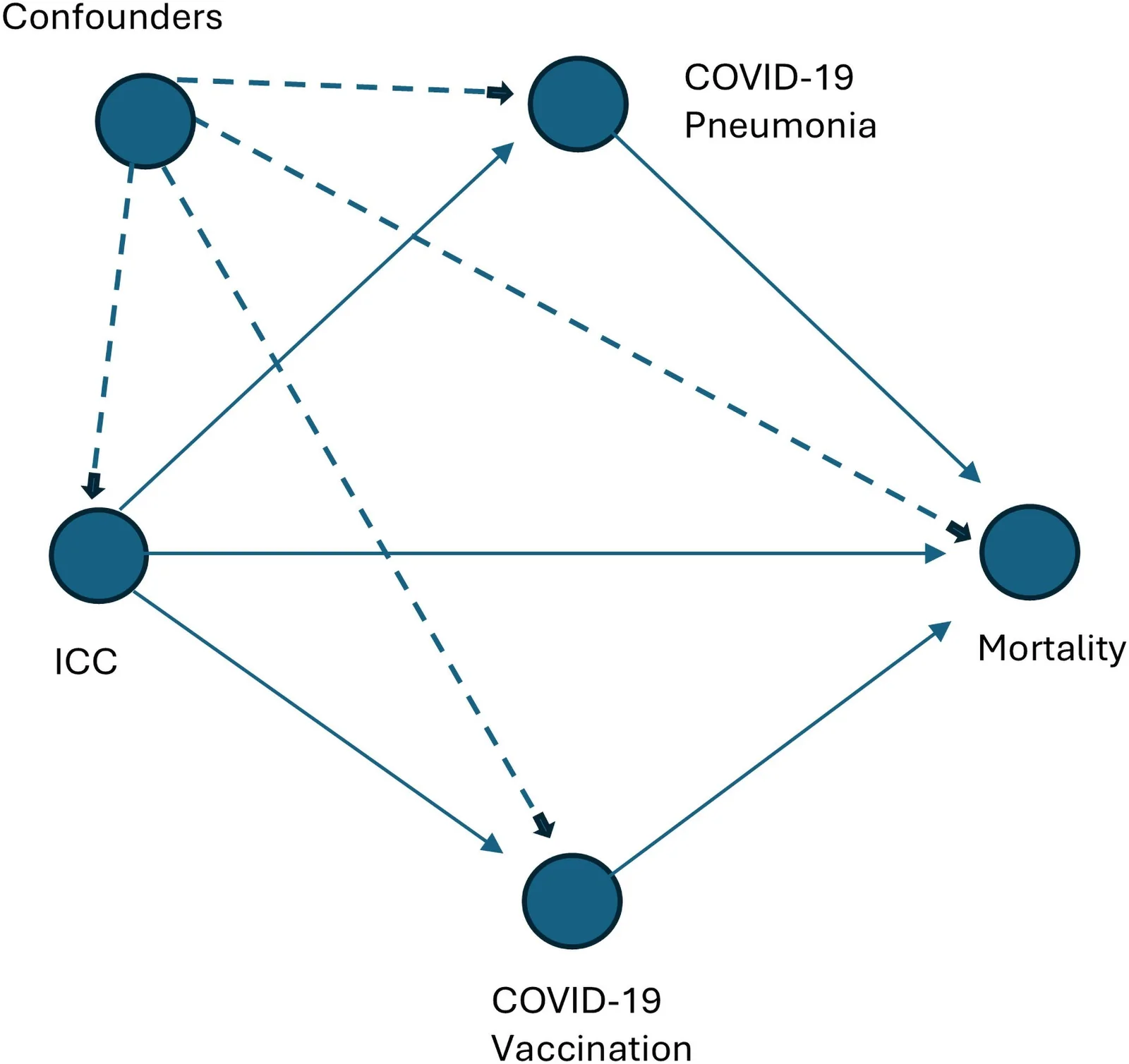
A basic causal diagram of immunocompromising conditions (ICCs), COVID-19 pneumonia, COVID-19 vaccination, status and mortality.

Data were collected for age and biological sex using an electronic case report form. Comorbidities associated with increased risk of mortality or ICCs were also collected including cancer, cardiac or cerebrovascular disease, chronic kidney disease, chronic liver disease, chronic lung disease, diabetes (type 1 or 2), hypertension, other immunocompromised states, neurologic conditions, and obesity. Figure 1 shows our assumptions regarding the causal structure of the data.

### Statistical Analysis

Descriptive analyses were used to describe baseline characteristics of participants: age, sex at birth, ethnicity, comorbidities, SARS-CoV-2 variant, COVID-19 pneumonia, and COVID-19 vaccination, overall and after stratifying by immunocompetent and immunocompromised status. Prevalence of immunocompromised individuals (with 95% confidence intervals [CIs], calculated using the Clopper–Pearson exact formula [21]) by year of hospital admission were estimated overall and stratified by ICC type (SOT, HM without HSCT, HM with HSCT, and both HM and SOT).

Logistic regression was used to estimate the overall effect of ICCs on 28-day in-hospital mortality. To evaluate whether the risk of death associated with ICCs has changed over time, we performed an interaction test. Regardless of the *P* value associated with this test, we also showed results of the main analysis after stratification by variant. Multivariable models included age, biological sex, clinical site, comorbidities, year of hospitalization, and variant as they were all on open confounding paths (described as a single node called “confounders”; Figure 1). In contrast, both COVID-19 pneumonia and vaccination were assumed to be on the causal pathways between ICCs and mortality. Indeed, participants with ICCs are (1) more likely to experience COVID-19 pneumonia and (2) have been prioritized to receive COVID-19 vaccinations. Standard adjustment for these factors, as done previously [16, 17], may at best estimate the indirect effect of ICCs, which does not go through these pathways, or in the worst-case scenario, introduce collider bias [22].

Under these assumptions, we employed separate counterfactual mediation analysis (one for each of the hypothesized intermediates) in the multiplicative scale, using odds ratio (OR) as estimand, to decompose the total effect (TE) of ICCs on the risk of mortality, in direct effect and indirect effects through the mediators. For this purpose, we used Stata (version 19.0) command “mediate” to estimate the OR for the natural direct effect (NDE) and the natural indirect effect (NIE), as well as the proportion mediated. In addition, we also estimated the controlled direct effect (CDE) of ICCs under the scenario that the whole cohort was to experience COVID-19 pneumonia during hospitalization versus no one did. We repeated this analysis with vaccination status as the mediator. Also, for these analyses, we present the overall results and those after stratification by variant when possible.

For the primary analysis of the TE of ICCs on mortality risk, we performed several sensitivity analyses. First, we investigated if different types of ICCs (SOT, HM with HSCT, and HM without HSCT) have different mortality risk profiles. Second, since we did not have reason for hospital discharge, and patients could have either recovered from infection or be transferred to another hospital due to worsening condition, we considered hospital discharge as an informative censoring mechanism and competing event of death using Fine–Gray subdistribution hazard models. Third, we also performed all analyses after removing individuals with other, less severe, ICCs. Fourth, as data from Sweden (6 acute care hospitals in Stockholm County [KI]) comprised 62% of our population, we performed the analysis including only participants from KI and the opposite, among participants not from KI. Finally, in the mediation analysis with vaccination as the intermediate, we used at least 3 doses of COVID-19 vaccine to define fully protected individuals (leaving those with 0–1 doses as the comparator) as well accounted for time elapsed since last vaccine dose.

While we report ORs, the cumulative incidence function, and subdistribution hazard ratios (sHRs) to maintain statistical consistency with our multivariable models, we use the term “risk” in our qualitative discussion to align with common clinical language and enhance interpretability. This approach is further justified by the low incidence of mortality in our population (10%), as the OR reliably approximates the risk ratio when the outcome is rare (<10%) [23].

All analyses were performed using Stata 19.0 software (StataCorp LLC, College Station, TX, USA).

## RESULTS

### Study Population

A total of 44 170 individuals were initially included in the analysis. Two sites (Alupe Sub-County Hospital in Busia, Kenya, and Hospital Regional de Alta Especialidad “Dr. Juan Graham Casasus” in Villahermosa, Mexico) reported no individuals with ICCs and were subsequently removed, leaving 42 488 individuals, with 1675 (3.9%) of them defined as having an ICC. The breakdown of ICCs was as follows: 799 (1.9%) HM without HSCT, 159 (0.4%) HM with HSCT, 707 (1.7%) with SOT, and 10 (<0.1%) with both HM and SOT. Fifty-five percent of our population was male, and the median age was 67 (interquartile range [IQR], 52–79) years. Those with ICCs tended to have more vaccinations (*P* < .001) and comorbidities (*P* < .001), including COVID-19 pneumonia (*P* < .001) (Table 1). Forty percent were diagnosed with wild-type strain, 19% with Alpha, 9% with Delta, and 31% with Omicron variants (18% BA.1, 5% BQ.1, 4% BA.5, 3% BA.2, <1% BA.4, <1% CH.1).

**Table 1.** Baseline Characteristics of Individuals Classified by Immunosuppressive Status in the EuCARE-HOSPITALIZED Study

Characteristic	No ICC (n = 42 495)	%	ICC (n = 1675)	%	Overall (N = 44 170)	%	*P* Value
Center^a^							
ASST	3756	9	13	1	3769	9	<.001
CHLO	757	2	42	3	799	2	
HHU	2052	5	300	18	2352	5	
*HRAE*	*932*	*2*	*0*	*0*	*932*	*2*	
IMPERIAL-Poole	1590	4	16	1	1606	4	
*KEMRI*	*750*	*2*	*0*	*0*	*750*	*2*	
KI^b^	25 549	60	1165	70	26 714	61	
PUM	4279	10	60	4	4339	10	
UNITOV	1158	3	8	1	1166	3	
VULSK	1672	4	71	4	1743	4	
	n = 40 813		n = 1675		n = 42 488		
Sex							
Female	18 425	45	600	36	19 025	45	<.001
Male	22 388	55	1075	64	23 463	55	
Age, y, median (IQR)	67 (55–76)		67 (52–80)		67 (52–79)		.0479
Comorbidities							
Cancer	2959	7	905	54	3864	9	<.001
Cardiac or cerebrovascular disease	11 706	29	586	35	12 292	29	<.001
Chronic kidney disease	4649	11	707	42	5356	13	<.001
Chronic liver disease	1073	3	91	5	1164	3	<.001
Chronic lung disease	6558	16	218	13	6776	16	<.001
Diabetes	8274	20	410	25	8684	20	<.001
Hypertension	17 508	43	983	59	18 491	44	<.001
Other immunosuppression	3240	8	0	0	3240	8	<.001
Neurological condition	3707	9	69	4	3776	9	<.001
Obesity	7099	17	207	12	7306	17	<.001
Variant							
Wild-type	16 513	41	432	26	16 945	40	<.001
Alpha	8000	20	178	11	8178	19	
Delta	3692	9	131	8	3823	9	
Omicron	12 409	30	932	56	13 341	31	
Other/unknown	199	1	2	0	201	1	
No. of vaccine doses received							
0	29 009	71	631	38	29 640	70	<.001
1	921	2	47	3	968	2	
2	3204	8	209	13	3413	8	
≥3	6923	17	743	44	7666	18	
Missing	756	2	45	3	801	2	
COVID-19 pneumonia	19 310	47	864	52	20 174	48	<.001
ICU admission	3987	10	177	11	4164	10	<.001
In-hospital mortality	4167	10	177	11	4344	10	<.001

Abbreviations: COVID-19, coronavirus disease 2019; ICU, intensive care unit; IQR, interquartile range.

^a^ASST, San Paolo Hospital and San Carlo Hospital; CHLO, Hospital de Santa Cruz, Hospital Egas Moniz, Hospital São Francisco Xavier; HHU, Düsseldorf University Hospital in Düsseldorf, Germany; HRAE, Hospital Regional de Alta Especialidad “Dr. Juan Graham Casasus” in Villahermosa, Mexico; IMPERIAL-Pool, Poole Hospital in Poole, United Kingdom; KEMRI, Alupe Sub-County Hospital in Busia, Kenya; KI, 6 acute care hospitals (Danderyd Hospital, Karolinska University Hospital Huddinge, Karolinska University Hospital Solna, Norrtälje Hospital, Södersjukhuset, and Södertälje Hospital) in Stockholm County, Sweden; PUM, PUM District Hospital in Szczecin, Poland; UNITOV, Hospital Tor Vergata Roma in Rome, Italy; VULSK, VULSK hospital in Vilnius, Lithuania. Sites marked in italics reported no individuals with ICCs and were removed from subsequent analyses.

^b^For sites in Sweden, data were linked using personal identification numbers, unique for each Swedish resident, from the TakeCare Intelligence database, the Stockholm regional healthcare data warehouse, Statistics Sweden, SmiNet, the National Vaccination Register, and the Swedish Intensive Care Registry.

### Prevalence at Hospital Admission Over Time

**Figure 2.**
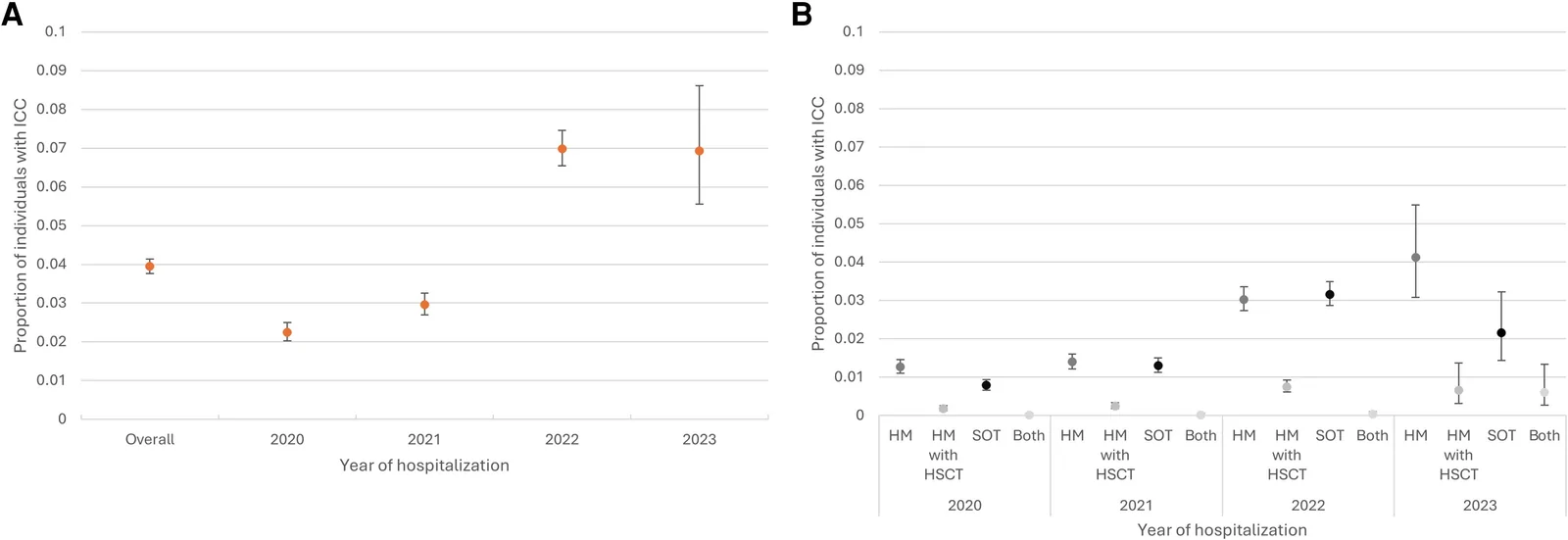
Proportion (95% CIs) of individuals hospitalized with COVID-19 with (*A*) any immunocompromising condition (ICC), and (*B*) ICCs stratified on hematological malignancies (HMs) with and without hematopoietic stem cell transplantation (HSCT), solid organ transplant (SOT), and both HM and SOT over the pandemic in the EuCARE-HOSPITALIZED cohort.

The proportion of ICCs was 2.2% (95% CI, 2.0%–2.5%) in participants admitted to hospital in 2020 and rose to 6.9% (95% CI, 5.6%–8.6%) by 2023 (Figure 2). A general increase in prevalence was observed for both HM and SOT (Figure 2).

### Risk of Mortality

Overall, 4344 (10.2%) individuals died in hospital within 28 days; 3023 (7.1%) individuals were censored having not been discharged within 28 days, and 35 121 (82.7%) were censored when they were discharged prior to 28 days.

**Figure 3.**
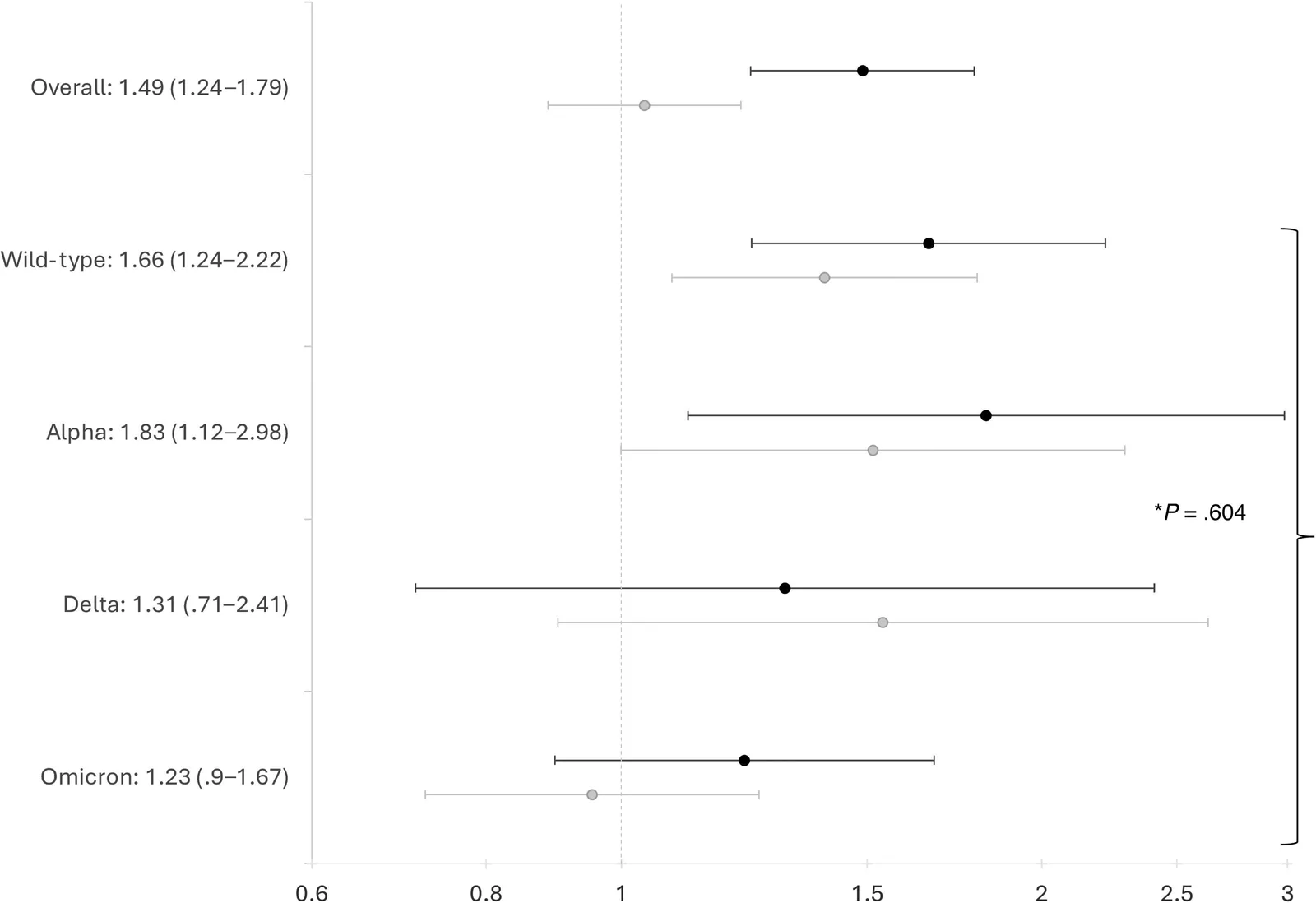
Results from multivariable logistic regression analysis on risk of 28-day in-hospital mortality (measured by odds ratios and 95% CIs) comparing those with immunocompromising conditions (ICCs) to those without ICCs, overall and by variant in the EuCARE-HOSPITALIZED cohort. *P value for interaction between ICC and variant. Estimates in gray are from unadjusted models, and estimates in black are adjusted for age, sex, center, and comorbidities; the overall model was additionally adjusted for year of hospitalization (fitted as a spline with 4 knots at 1 July 2020, 1 September 2021, 1 October 2021, and 9 March 2022 to coincide with the data on dexamethasone and the Omicron surge in Europe and variant).

**Figure 4.**
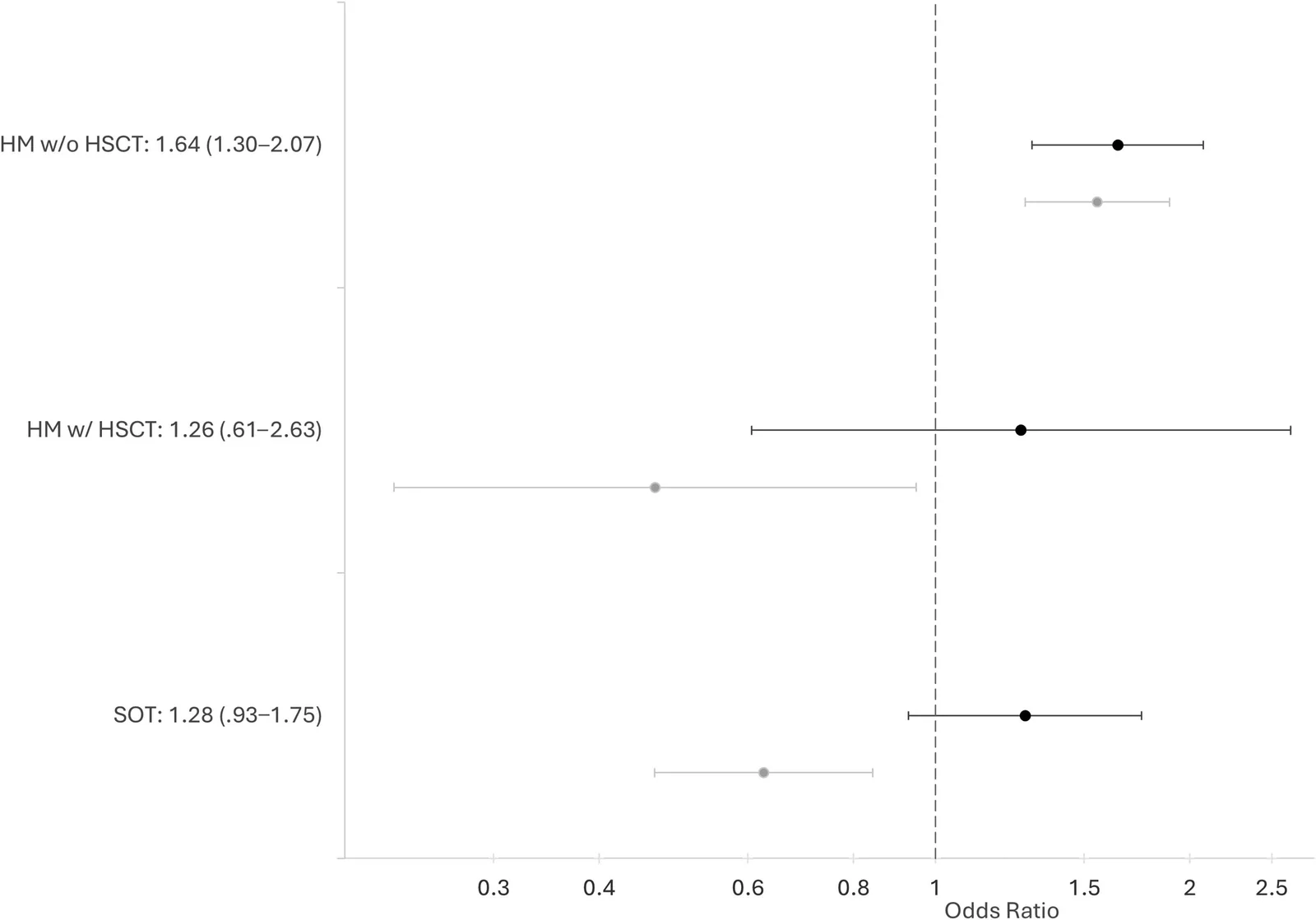
Results from multivariable logistic regression analysis on risk of 28-day in-hospital mortality (measured by odds ratios and 95% CIs) comparing those with hematological malignancies (HMs) without hematopoietic stem cell transplantation (HSCT), those with HM and HSCT, and those with solid organ transplant (SOT) compared to immunocompetent individuals in the EuCARE-HOSPITALIZED cohort. Estimates in gray are from unadjusted models; estimates in black are adjusted for age, sex, center, comorbidities, and year of hospitalization (fitted as a spline with 4 knots at 1 July 2020, 1 September 2021, 1 October 2021, and 9 March 2022 to coincide with the data on dexamethasone and the Omicron surge in Europe and variant).

Using logistic regression to assess the risk of 28-day in-hospital mortality, we found an increased odds among those with ICCs versus immunocompetent between 2020 and 2023 (OR, 1.49 [95% CI, 1.25–1.79]; Figure 3). We found similar results after restricting the analysis to participants enrolled at the KI centers (OR, 1.92 [1.53–2.39]; Supplementary Figure 1). Results were also similar when we performed the competing risk Cox survival analysis (sHR, 1.43 [95% CI, 1.22–1.69]; *P* < .001; Supplementary Figure 2) and when removing individuals with other immunosuppressive conditions (Supplementary Figure 3). When stratifying by ICC category, we found that individuals with HM without HSCT versus immunocompetent had the highest odds of mortality (OR, 1.64 [95% CI, 1.30–2.07]), followed by SOT and HM with HSCT (OR, 1.28 [95% CI, .93–1.75] and 1.26 [95% CI, .61–2.63], respectively) (Figure 4).

The odds of mortality associated with ICCs was the highest during the wild-type phase and appeared to be slightly attenuated with Omicron (adjusted OR [aOR], 1.23 [95% CI, .90–1.67]). However, the analysis showed little evidence that SARS-CoV-2 variants were effect measure modifiers for this association (interaction *P* = .60; Figure 3).

**Figure 5.**
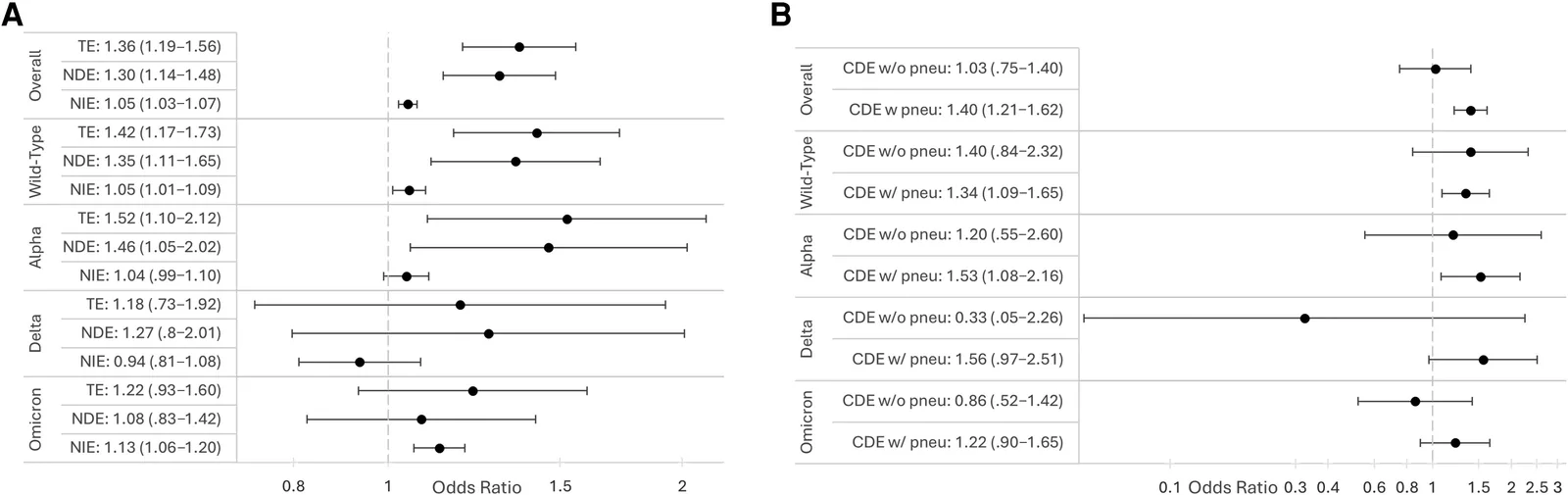
Mediation model for the (*A*) total effect (TE), natural direct effect (NDE), and natural indirect effect (NIE) and (*B*) controlled direct effect (CDE) of immunocompromising conditions on mortality among those with and without COVID-19 pneumonia (pneu) in the EuCARE-HOSPITALIZED cohort overall and stratified by variant. Models are adjusted for age, sex, center, and comorbidities; the overall model was additionally adjusted for year of hospitalization (fitted as a spline with 4 knots at 1 July 2020, 1 September 2021, 1 October 2021, and 9 March 2022 to coincide with the data on dexamethasone and the Omicron surge in Europe and variant).

Mediation analysis was conducted to assess whether the effect of ICC on in-hospital mortality was mediated through COVID-19 pneumonia and vaccination. For the estimate of the TE, the analysis almost exactly replicated what we found with the standard logistic regression model (aOR, 1.36 [95% CI, 1.19–1.56]). The NDE was similar to the TE (OR, 1.30 [95% CI, 1.14–1.48]), indicating that most of the effect of ICCs on mortality was through its direct effect. Consistently, the NIE through pneumonia was small (OR, 1.03 [95% CI, 1.03–1.07]), corresponding to a proportion of the total ICCs effect mediated by pneumonia of only 17% (95% CI, 8%–27%) (Figure 5). These results were consistent in the wild-type, Alpha, and Delta variants, although NIE was slightly elevated in the Omicron era (OR, 1.13 [95% CI, 1.06–1.20]) with a proportion mediated of 63% (Figure 5).

We then estimated the CDE of ICCs on mortality at 2 fixed levels of pneumonia status (participants with and without pneumonia). In this analysis, we found that the CDE under the counterfactual assumptions of everybody experiencing pneumonia versus nobody experiencing pneumonia with a wild-type virus during hospitalization was similar (CDE without pneumonia: OR, 1.40 [95% CI, .84–2.32] and CDE with pneumonia: OR, 1.34 [95% CI, 1.09–1.65]). However, during the Omicron phase, ICCs appeared to increase mortality risk only under the scenario in which everybody had experienced COVID-19 pneumonia (CDE: OR, 1.22 [95% CI, .90–1.65]; Figure 5).

**Figure 6.**
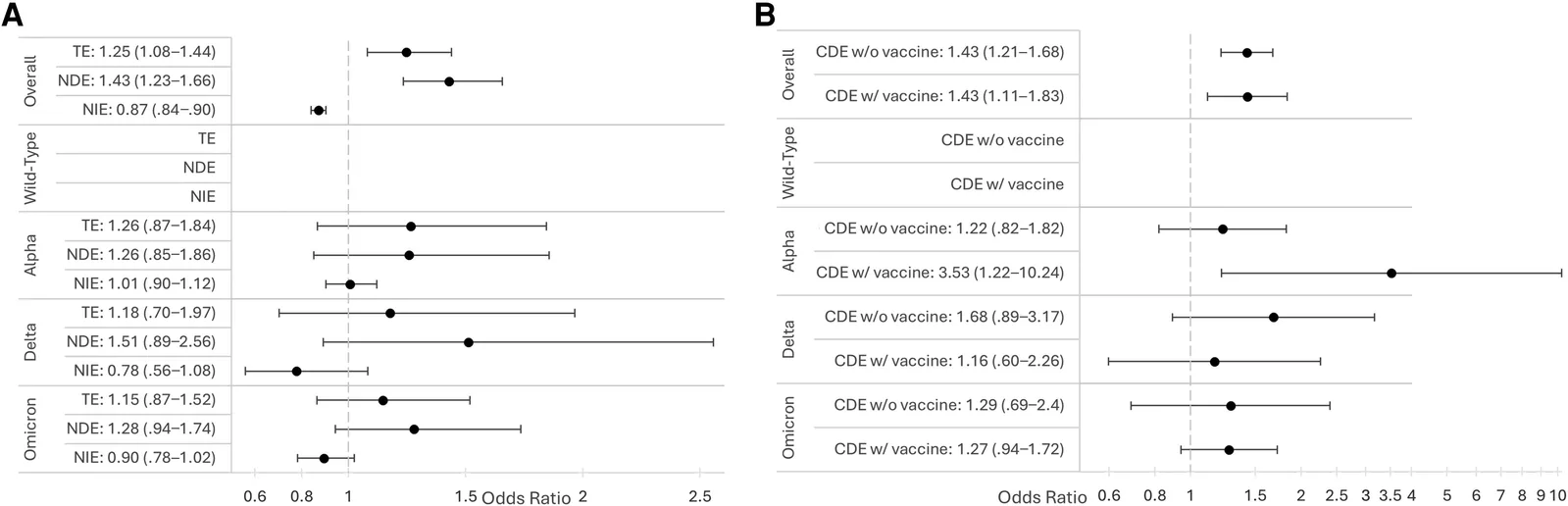
Mediation model for (*A*) the total effect (TE), natural direct effect (NDE), and natural indirect effect (NIE) and (*B*) controlled direct effect (CDE) of immunocompromising conditions on mortality among those with and without COVID-19 vaccination in the EuCARE-HOSPITALIZED cohort overall and stratified by variant. Models are adjusted for age, sex, center, and comorbidities; the overall model was additionally adjusted for year of hospitalization (fitted as a spline with 4 knots at 1 July 2020, 1 September 2021, 1 October 2021, and 9 March 2022 to coincide with the data on dexamethasone and the Omicron surge in Europe and variant).

A mirrored approach was used to evaluate how much of the TE of ICC on in-hospital mortality may be mediated by COVID-19 vaccination. The TE of ICC on mortality was 1.25 (95% CI, 1.08–1.44). Interestingly, in this analysis the OR for the NIE through vaccination status was <1 (OR, 0.87 [95% CI, .84–.90]), suggesting that, on average, receiving vaccination resulted in a decreased risk of death. Indeed, the direct effect of ICCs bypassing vaccination was larger than the TE in this analysis (OR, 1.43 [95% CI, 1.23–1.66]; Figure 6). Although proportion mediated cannot be calculated if the mediator is not a risk factor, the results indicate moderate mediation by vaccination. Results were also consistent across SARS-CoV-2 variant strata. Of note, the wild-type variant stratum is missing for this analysis as vaccine was not available during this early phase of the pandemic, resulting in too few vaccinated participants (Figure 6). In a sensitivity analysis comparing those vaccinated with 0 or 1 dose to those with 3 or more doses, we found similar results to those of the main analysis (TE: OR, 1.24 [95% CI, .93–1.66]; NDE: OR, 1.40 [95% CI, 1.02–1.92]; NIE: OR, 0.88 [95% CI, .75–1.04]) (Supplementary Figure 4). This sensitivity analysis was performed only in the Omicron era as there were too few individuals with 3 or more vaccinations in all other pandemic waves. Similarly, when comparing those unvaccinated or who received a maximum of 1 dose to those with 2 or more doses, the last of which was within 6 months prior to the hospitalization, we found similar results (TE: OR, 1.22 [95% CI, 1.06–1.41]; NDE: OR, 1.30 [95% CI, 1.12–1.50]; NIE: OR, 0.94 [95% CI, .92–.97]) (Supplementary Figure 5).

Overall, the CDE analysis for vaccination, evaluating the direct effect of ICCs under the counterfactual scenarios of nobody undergoing vaccination versus everybody undergoing vaccination carried almost identical results (Figure 6). Results were also similar regardless of variants. This was also somewhat expected as participants had to be hospitalized to be included in the analysis suggesting that response to vaccination, albeit somewhat protective, had been suboptimal in people with ICCs.

## DISCUSSION

Our analysis shows that, although the COVID-19 pandemic has evolved into variants with a lower degree of pathogenicity [2, 22], with increased availability of targeted treatments and increased vaccination coverage, during the Omicron wave, severely immunocompromised individuals appear to remain at higher risk of in-hospital mortality compared with immunocompetent individuals. Our sensitivity analysis using competing risk and excluding individuals with other ICCs supports the robustness of our findings. These analyses tried to account for potential biases due to informative censoring and heterogeneity within the ICC population.

Thus, the first important take-home message for our work is that, despite the fact that the World Health Organization declared the end of the public health emergency in 2023 [1], our findings underscore the continued vulnerability of ICC patients with HMs and SOTs, supporting previous findings [9, 24].

The prevalence of ICCs at time of hospital admission increased steadily from 2020 to 2023, likely reflecting relaxed preventive measures during Omicron and greater exposure among vulnerable groups. In early phases of the pandemic individuals with immunosuppressive conditions would have avoided hospitals unless essential. Additionally, with the availability of approved antiviral treatments in more recent waves, people with ICCs were also more likely to be admitted to hospital for elective treatment in the first 5–7 days after symptom onset in order to receive early antiviral therapy with remdesivir [25] and/or monoclonal antibodies such as casirivimab/imdevimab or sotrovimab [26, 27], which were only rarely used in an outpatient settings. Given the drug interactions between ritonavir and immunosuppressive drugs or oncological therapies, the option of early outpatient antiviral therapy with nirmatrelvir/ritonavir [28] was often not available for this patient group. Additionally, because we observe less severe disease and death during Omicron [2, 29], people without ICCs were less seriously affected. In contrast, those with ICCs could have been disproportionately affected and admitted to hospital during this era. The apparent lower pathogenicity of Omicron may be confounded by high levels of preexisting immunity due to vaccination and previous SARS-CoV-2 exposure [30]. Even then, ICC status was associated with an overall 49% increased risk of 28-day in-hospital mortality as compared to immunocompetence. This elevated risk persisted across all pandemic waves (there was no evidence that variants was an effect measure modifier), showing that individuals’ underlying immunosuppressive conditions made them more vulnerable regardless of variant.

Mediation analysis showed that COVID-19 pneumonia explained only 17% of the ICC-related mortality, suggesting that most risk stems from immunocompromised status itself. Notably, the analysis also showed that, on the other hand, pneumonia accounted for 63% of the TE during Omicron, possibly reflecting later presentation, delayed treatment, and poorer prognosis in ICC patients admitted with pneumonia.

Conversely, vaccination status appeared to be a stronger mediator. Indeed, the data show that receiving vaccination because of preexisting ICCs resulted in a decreased risk of death. This reinforces the importance of prioritizing individuals with ICCs for vaccination and booster programs, as suggested by others [31, 32].

Several limitations of this study should be acknowledged. First, the observational nature of our study limits the causal interpretability of our findings, and our results rely on the untestable assumption of no unmeasured residual confounding, including the key assumption of no mediator-outcome confounding. We are aware that more complicated methods exist to further decompose between what is mediation from what is interaction which have not been employed here [33]. It seemed an unnecessary complication as none of our analyses carried evidence that there was strong mediation through the hypothesized intermediates. Second, we lacked detail on the type and exact timing of immunosuppressive therapies, which may influence disease severity, immune function, and vaccine response. Third, the reason for hospital discharge was not available. We tried to account for this by performing a competing risk analysis, but there could be residual collider bias from informative censoring. Fourth, most of the data were collected in a single EuCARE participating site (KI); however, the analysis conducted on data provided by non-KI sites showed overlapping CIs with those of our main analysis. Finally, we used a broad definition for the mediator, COVID-19 pneumonia. Due to data constraints, pneumonia was defined by the requirement for any new supplemental oxygen therapy during hospitalization. While this ensures the inclusion of patients with clinically relevant respiratory impairment, it likely encompasses a wide spectrum of respiratory pathology, ranging from mild hypoxemia to more severe consolidation. The high prevalence of this mediator in our cohort (∼50%) suggests that our findings reflect the overall impact of COVID-19–related respiratory support rather than a specific pneumonia phenotype. Future studies with more granular data on respiratory support levels, such as high-flow oxygen or mechanical ventilation, are needed to refine these mediation pathways and determine if the effect size varies across different severities of lung injury.

Our analysis also has several strengths. First is the very strict and accurate definition of severe immunosuppression status based on ICD-10 codes. Also, the sample size and study duration encompassed people with ICCs recruited across all of Europe and covered several waves of the COVID-19 pandemic. Of course, although a small proportion of participants were infected with variants circulating after October 2022 (eg, BQ.1/BQ.1.1), we cannot be sure the results will be consistent and applicable to more recent waves of the Omicron BA.2.86 family. However, evidence suggest that there was very little change in pathogenicity with these more recent strains [34, 35].

In conclusion, our findings highlight the need for sustained surveillance, therapeutic interventions, and prioritization for vaccinations to reduce mortality in individuals with severe immunocompromise. Future research should explore the role of specific immunosuppressive therapies, vaccine boosting efficacy, and post-discharge outcomes to better inform clinical management.

## Supplementary Material

jiag254_Supplementary_Data

## Supplementary Data

Supplementary materials are available at *The Journal of Infectious Diseases* online (http://jid.oxfordjournals.org/). Supplementary materials consist of data provided by the author that are published to benefit the reader. The posted materials are not copyedited. The contents of all supplementary data are the sole responsibility of the authors. Questions or messages regarding errors should be addressed to the author.
