# Supplementary material for: Increased In-Hospital Mortality in Immunocompromised Individuals Hospitalized With COVID-19 During the Global Pandemic: A Multinational Cohort Study in the EuCARE Project
Source: J Infect Dis. 2026 May 8;234(3):e496–505. doi: 10.1093/infdis/jiag254 (PMC13600395; doi:10.1093/infdis/jiag254)
Supplement: Supplementary file 1 [file jiag254_supplementary_data.docx]

# Supplementary Materials:

## Definitions for ICCs

Whether participants carried other less severe immunocompromised conditions was also recorded, but this information was not used to define the exposure of interest. ICCs were assumed to pre-exist hospital entry and had to be diagnosed within 12 months of SARS-CoV-2 diagnosis, or the patient had to be receiving treatment for these chronic conditions in the 12 months preceding their SARS-CoV-2 hospitalization.

## Variants

For a proportion of participants (15.5%), SARS-CoV-2 variant was classified using sequencing. If sequencing data was not available, the variant was inferred from the 7-day moving average national distribution of variants retrieved from the Global Initiative on Sharing All Influenza Data (GISAID), as we have done previously^1^.

### Supplementary Figure 1: Odds Ratio for the risk of mortality among immunocompromised conditions (ICC) compared to no ICC among those in Sweden (KI) and among those not in KI.

|  | Odds Ratio |
| --- | --- |
| KI: 1.92 (1.54, 2.39) | 1.02609 |
| non KI: 0.96 (0.67, 1.38) | 1.39763 |

|  | or |
| --- | --- |
| 1.91501 | 1.5 |
| 0.959987 | 0.5 |

|  | y=0 |
| --- | --- |
| 0.1 | 0 |
| 0.3 | 0 |
| 0.4 | 0 |
| 0.6 | 0 |
| 0.8 | 0 |
| 1 | 0 |
| 1.5 | 0 |
| 2 | 0 |
| 2.5 | 0 |
| 3 | 0 |
| 3 | 0 |
| 3.5 | 0 |
| 4 | 0 |
| 5 | 0 |
| 6 | 0 |
| 7 | 0 |

|  | x=1 |
| --- | --- |
| 1 | 5 |
| 1 | 4.5 |
| 1 | 3.5 |
| 1 | 2.5 |
| 1 | 1.5 |
| 1 | 0.5 |
| 1 | 0 |

Due to decreased sample size, we could not stratify the analyses by variant, so the above results include all pandemic waves. Models were adjusted for variant, year of hospitalization (fitted as a spline with 4 knots at 1 July 2020, 1 sept 2021, 1 oct 2021 and 9 March 2022 to coincide with the data on dexamethasone and the omicron surge in Europe), age, gender and comorbidities.

### Supplementary Figure 2: Results from the competing-risk survival analysis: subhazard ratios for the risk of mortality among immunocompromising conditions (ICC) compared to no ICC in the EuCARE HOSPITALIZED cohort.

*p-value for variant and ICC interaction.
Estimates in grey are from unadjusted models, estimates in black are adjusted for age, gender, centre and comorbidity, the overall model was additionally adjusted for, year of hospitalization (fitted as a spline with 4 knots at 1 July 2020, 1 sept 2021, 1 oct 2021 and 9 March 2022 to coincide with the data on dexamethasone and the omicron surge in Europe and variant).

|  | label |
| --- | --- |
| Overall: 1.43 (1.22, 1.69) | 1.43324 |
| Wild-Type: 1.55 (1.21, 1.98) | 1.5475 |
| Alpha: 1.7 (1.14, 2.52) | 1.69635 |
| Delta: 1.36 (0.79, 2.34) | 1.36092 |
| Omicron: 1.21 (0.90, 1.62) | 1.20839 |

|  | shr |
| --- | --- |
| 1.43324 | 4.6 |
| 1.5475 | 3.6 |
| 1.69635 | 2.6 |
| 1.36092 | 1.6 |
| 1.20839 | 0.6 |

|  | y=0 |
| --- | --- |
| 0.1 | 0 |
| 0.3 | 0 |
| 0.4 | 0 |
| 0.6 | 0 |
| 0.8 | 0 |
| 1 | 0 |
| 1.5 | 0 |
| 2 | 0 |
| 2.5 | 0 |
| 3 | 0 |
| 3 | 0 |
| 3.5 | 0 |
| 4 | 0 |
| 5 | 0 |
| 6 | 0 |
| 7 | 0 |

|  | x=1 |
| --- | --- |
| 1 | 5.5 |
| 1 | 4.6 |
| 1 | 3.6 |
| 1 | 2.6 |
| 1 | 1.6 |
| 1 | 0.6 |
| 1 | 0 |

|  | unadjusted |
| --- | --- |
| 1.02592 | 4.4 |
| 1.33825 | 3.4 |
| 1.42407 | 2.4 |
| 1.48908 | 1.4 |
| 0.948835 | 0.4 |

### Supplementary Figure 3: Odds ratios for the risk of mortality among immunocompromising conditions (ICC) compared to no ICC among only those without other immunocompromising conditions in the EuCARE HOSPITALIZED cohort.

|  |  |
| --- | --- |
| Overall: 1.5 (1.24, 1.81) | 1.49844 |
| Wild-Type: 1.65 (1.23, 2.22) | 1.65327 |
| Alpha: 1.81 (1.10, 2.98) | 1.81103 |
| Delta: 1.31 (0.71, 2.43) | 1.31304 |
| Omicron: 1.25 (0.91, 1.72) | 1.25129 |

|  | adjusted |
| --- | --- |
| 1.49844 | 4.5 |
| 1.65327 | 3.5 |
| 1.81103 | 2.5 |
| 1.31304 | 1.5 |
| 1.25129 | 0.5 |

|  |  |
| --- | --- |
| 0.1 | 0 |
| 0.3 | 0 |
| 0.4 | 0 |
| 0.6 | 0 |
| 0.8 | 0 |
| 1 | 0 |
| 1.5 | 0 |
| 2 | 0 |
| 2.5 | 0 |
| 3 | 0 |
| 3 | 0 |
| 3.5 | 0 |

|  | x=1 |
| --- | --- |
| 1 | 5.5 |
| 1 | 4.6 |
| 1 | 3.6 |
| 1 | 2.6 |
| 1 | 1.6 |
| 1 | 0.6 |
| 1 | 0 |

Models are adjusted for age, gender, centre and comorbidity, the overall model was additionally adjusted for, year of hospitalization (fitted as a spline with 4 knots at 1 July 2020, 1 sept 2021, 1 oct 2021 and 9 March 2022 to coincide with the data on dexamethasone and the omicron surge in Europe and variant).

### Supplementary Figure 4: Mediation model for a) the total effect (TE), natural direct effect (NDE), natural indirect effect (NIE) and b) controlled direct effect (CDE) of ICC on mortality among those with and without at least 3 COVID-19 vaccinations in the EuCARE HOSPITALIZED cohort

|  |  |
| --- | --- |
| TE: 1.24 (0.93, 1.66) | 1.23962 |
| NDE: 1.4 (1.02, 1.92) | 1.40269 |
| NIE: 0.88 (0.75, 1.04) | 0.883744 |
| CDE w/o vaccine: 1.26 (0.68, 2.34) | 1.26486 |
| CDE w/ vaccine: 1.49 (1.09, 2.03) | 1.48867 |

|  |  |
| --- | --- |
| 1.24629 | 4.5 |
| 1.42988 | 3.5 |
| 0.871606 | 2.5 |
| 1.42516 | 1.5 |
| 1.42879 | 0.5 |

|  |  |
| --- | --- |
| 0.1 | 0 |
| 0.3 | 0 |
| 0.4 | 0 |
| 0.6 | 0 |
| 0.8 | 0 |
| 1 | 0 |
| 1.5 | 0 |
| 2 | 0 |
| 2.5 | 0 |
| 3 | 0 |
| 3 | 0 |
| 3.5 | 0 |
| 4 | 0 |
| 5 | 0 |
| 6 | 0 |
| 7 | 0 |

|  |  |
| --- | --- |
| 1 | 5 |
| 1 | 4.5 |
| 1 | 3.5 |
| 1 | 2.5 |
| 1 | 1.5 |
| 1 | 0.5 |
| 1 | 0 |

Models adjusted for calendar year, age, gender, centre, year of hospitalization (fitted as a spline with 4 knots at 1 July 2020, 1 sept 2021, 1 oct 2021 and 9 March 2022 to coincide with the data on dexamethasone and the omicron surge in Europe (in overall models), and comorbidities. This model only includes data in the Omicron era, as there were too few individuals with 3 or more vaccinations in all other pandemic waves.

### Supplementary Figure 5: Mediation model for a) the total effect (TE), natural direct effect (NDE), natural indirect effect (NIE) and b) controlled direct effect (CDE) of ICC on mortality among those with and without at least two COVID-19 vaccinations, with the most recent vaccine administered within 6 months prior to hospitalisation in the EuCARE HOSPITALIZED cohort

|  |  |
| --- | --- |
| TE: 1.22 (1.06, 1.41) | 1.22416 |
| NDE: 1.3 (1.12, 1.5) | 1.29836 |
| NIE: 0.94 (0.92, 0.97) | 0.942852 |
| CDE w/o vaccine*: 1.26 (1.08, 1.47) | 1.25669 |
| CDE w/ recent vaccine*: 1.72 (1.27, 2.33) | 1.72272 |

|  |  |
| --- | --- |
| 1.22416 | 4.5 |
| 1.29836 | 3.5 |
| 0.942852 | 2.5 |
| 1.25669 | 1.5 |
| 1.72272 | 0.5 |

|  |  |
| --- | --- |
| 0.1 | 0 |
| 0.3 | 0 |
| 0.4 | 0 |
| 0.6 | 0 |
| 0.8 | 0 |
| 1 | 0 |
| 1.5 | 0 |
| 2 | 0 |
| 2.5 | 0 |
| 3 | 0 |
| 3 | 0 |
| 3.5 | 0 |
| 4 | 0 |
| 5 | 0 |
| 6 | 0 |
| 7 | 0 |

|  |  |
| --- | --- |
| 1 | 5 |
| 1 | 4.5 |
| 1 | 3.5 |
| 1 | 2.5 |
| 1 | 1.5 |
| 1 | 0.5 |
| 1 | 0 |

* vaccine within 6 months prior to the date of hospitalisation

Models adjusted for calendar year, age, gender, centre, year of hospitalization (fitted as a spline with 4 knots at 1 July 2020, 1 sept 2021, 1 oct 2021 and 9 March 2022 to coincide with the data on dexamethasone and the omicron surge in Europe (in overall models), and comorbidities.

## Descriptions of centres and their data sources

**ASST:**

Data from ASST Santi Paolo and Carlo included two main hospitals (San Paolo Hospital and San Carlo Hospital) in Milan, Italy. Demographic, clinical and microbiological data were collected from the electronic health record (EHR) system of ASST Santi Paolo and Carlo. Data about vaccination were collected from the Socio-health information system of the Lombardy region for residents in the Lombardy region and from the EHR system of ASST Santi Paolo and Carlo for participants residing in other regions. Participants who were discharged alive either could be discharged to their home or to another hospital or to subacute centres, rehabilitation centres or long-term facilities.

**CHLO:**

Data from CHLO included data from three hospitals (Hospital de Santa Cruz, Hospital Egas Moniz, Hospital São Francisco Xavier) included in the CHLO group in Lisbon, Portugal. The SClínico platform aggregates informational data pertaining the following: demographics, place of birth, migration, drug prescriptions, International Statistical Classification of Diseases and Related Health Problems 10th Revision (ICD-10) codes, all microbiological tests and all notified polymerase chain reaction (PCR) severe acute respiratory syndrome coronavirus 2 (SARS-CoV-2) positive test results performed in participants attending either of the 3 CHLO hospitals in Lisbon. The Electronic Health Registry (RSE) included information regarding coronavirus disease 2019 (COVID-19) vaccinations per participant, administered in Portugal. Participants who were discharged alive either could be discharged to their home or to another hospital or care facility not covered by the data sources used.

**HHU:**

Data from HHU included data from all wards of the Düsseldorf University hospital in Düsseldorf, Germany. Data was extracted from the electronic health records of the hospital. Some information was additionally extracted manually from the patient files. Participants who were discharged alive either could be discharged to their home or to another hospital or care facility not covered by our data sources.

**HRAE:**

Data from HRAE came from Hospital Regional de Alta Especialidad “Dr. Juan Graham Casasus” in Villahermosa, Mexico. Data was obtained both from the EHR system and from health records on paper. Microbiological data and vaccination data were retrieved from national epidemiological registries and local systems. Participants who were discharged alive were discharged to their home since no other hospitals or centres had the capacity to care for COVID-19 patients in the region.

**IMPERIAL:**

Data from IMPERIAL came from Poole Hospital in Poole, United Kingdom. All clinical, patient and laboratory data was originally extracted from the hospital electronic medical records and additional medical notes and entered into electronic case report forms (CRFs). Data was submitted electronically to the International Severe Acute Respiratory and emerging Infection Consortium (ISARIC) Study Center Oxford who coded the patient’s identity and extracted a coded data file covering the retrospective dates. Whole genome sequencing data was linked manually to this file by the PI and re-anonymised. Participants who were discharged alive could be sent to home or residential care centre from the hospital.

**KEMRI:**

All data collected by KEMRI came from one ward in Alupe Sub-County Hospital in Busia, Kenya. All data was extracted from paper-based in-patient medical files. Inpatient identification numbers as indicated in respective inpatient files were linked to the assigned site study identification number. Discharge represented either participant testing negative after hospitalisation or participant recommended for home-based care.

**KI:**

Data from KI came from six acute care hospitals (Danderyd Hospital, Karolinska University Hospital Huddinge, Karolinska University Hospital Solna, Norrtälje Hospital, Södersjukhuset, and Södertälje Hospital) in Stockholm County, Sweden. Data were linked using personal identification numbers, unique for each Swedish resident, from the TakeCare® Intelligence database, the Stockholm regional healthcare data warehouse (VAL), Statistics Sweden, SmiNet, the National Vaccination Register (NVR), and the Swedish Intensive Care Registry (SIR). The TakeCare® Intelligence database included all microbiological tests performed in patients attending the six acute care hospitals. VAL contains data from healthcare databases within the Stockholm Region, including demographics, migration, drug prescriptions, and International Statistical Classification of Diseases and Related Health Problems 10th Revision (ICD-10) codes from all inpatient stays and outpatient visits reimbursed by Region Stockholm [1]. Data from Statistics Sweden were used to collect information on region of birth and education level [2]. SmiNet contained all PCR SARS-CoV-2 positive test results notified in accordance with the Communicable Diseases Act [3]. The data from NVR included all COVID-19 vaccinations administered in Sweden to the Stockholm County population [4]. SIR, a national intensive care units (ICU) in Sweden, contained all ICU admissions in Stockholm County [5]. Participants who were discharged alive could either be discharged to their home or to another hospital or care facility not covered by the data sources used.

**PUM:**

Data from PUM came from PUM District Hospital in Szczecin, Poland. Data were extracted using personal identification number (unique for every Polish patient) and internal hospital number (also unique for every patient) from the electronic patient health records. These records hold both medical history, follow-up data as well as laboratory data integrated for every case. The database also includes basic laboratory and microbiologic data, admission and discharge, including initial and discharge diagnosis coded based on ICD-10 codes. Vaccination data were extracted from national database on COVID-19 vaccination and linked to patient data. Sequence data were linked from national COVID-19 sequencing program and local sequencing program, which were also linked to the patient data. Discharge from the hospital represented the final discharge for all hospital wards. This meant that participants discharged alive could be discharged to their home, a care facility or another hospital.

**UNITOV:**

All data from UNITOV came from Hospital Tor Vergata Roma in Rome, Italy. Demographics, epidemiological, clinical and laboratory data were retrieved by extracting them both from the EHR systems and from the paper medical records of the hospital where patients were followed, according to the European Regulation on the protection of personal data n. 679/2016 and the Italian Legislative Decree 196/2003. Discharge from the hospital represented the final discharge for all hospital wards. This meant that participants discharged alive could be discharged to their home, a care facility or another hospital.

**VULSK**:

Data from VULSK came from the VULSK hospital in Vilnius, Lithuania. All data was extracted from the hospital electronic medical records. Discharge from the hospital represented the final discharge for all hospital wards. This meant that participants discharged alive could be discharged to their home, a care facility or another hospital.

1.	Hedberg P, Parczewski M, Serwin K, et al. In-hospital mortality during the wild-type, alpha, delta, and omicron SARS-CoV-2 waves: a multinational cohort study in the EuCARE project. *Lancet Reg Health Eur* 2024; **38**: 100855.
